# Dual Antigen T Cell Engagers Targeting CA9 as an Effective Immunotherapeutic Modality for Targeting CA9 in Solid Tumors

**DOI:** 10.3389/fimmu.2022.905768

**Published:** 2022-07-06

**Authors:** Nazanin Tatari, Xiaoyu Zhang, Shawn C. Chafe, Dillon McKenna, Keith A. Lawson, Minomi Subapanditha, Muhammad Vaseem Shaikh, Mathieu Seyfrid, Neil Savage, Chitra Venugopal, Jason Moffat, Sheila K. Singh

**Affiliations:** ^1^ Department of Biochemistry and Biomedical Sciences, McMaster University, Hamilton, ON, Canada; ^2^ Centre for Discovery in Cancer Research, McMaster University, Hamilton, ON, Canada; ^3^ Donnelly Centre, University of Toronto, Toronto, ON, Canada; ^4^ Department of Molecular Genetics, University of Toronto, Toronto, ON, Canada; ^5^ Department of Surgery, Faculty of Health Sciences, McMaster University, Hamilton, ON, Canada

**Keywords:** glioblastoma, immunotherapy, dual antigen T cell engagers, hypoxic niche, CA9, clear cell renal cell carcinoma

## Abstract

Glioblastomas (GBM), the most common malignant primary adult brain tumors, are uniformly lethal and are in need of improved therapeutic modalities. GBM contain extensive regions of hypoxia and are enriched in therapy resistant brain tumor-initiating cells (BTICs). Carbonic anhydrase 9 (CA9) is a hypoxia-induced cell surface enzyme that plays an important role in maintenance of stem cell survival and therapeutic resistance. Here we demonstrate that CA9 is highly expressed in patient-derived BTICs. CA9^+^ GBM BTICs showed increased self-renewal and proliferative capacity. To target CA9, we developed dual antigen T cell engagers (DATEs) that were exquisitely specific for CA9-positive patient-derived clear cell Renal Cell Carcinoma (ccRCC) and GBM cells. Combined treatment of either ccRCC or GBM cells with the CA9 DATE and T cells resulted in T cell activation, increased release of pro-inflammatory cytokines and enhanced cytotoxicity in a CA9-dependent manner. Treatment of ccRCC and GBM patient-derived xenografts markedly reduced tumor burden and extended survival. These data suggest that the CA9 DATE could provide a novel therapeutic strategy for patients with solid tumors expressing CA9 to overcome treatment resistance.

## Introduction

Glioblastoma (GBM), a highly aggressive astrocytic tumor (WHO grade IV), is the most common primary malignant brain tumor in adults (1, 2). Despite aggressive multi-modal treatment, including maximal safe surgical resection, chemotherapy with the alkylating agent temozolomide (TMZ) and radiotherapy, tumor re-growth and patient relapse occurs within 7-9 months post-diagnosis (3, 4). The average survival in GBM patients is only 12-14 months (5–9) with an abysmal two-year survival rate of 16.9% and only 5.5% of patients surviving at five years and 2.9% at ten years (4), underscoring the urgent need for novel therapeutic approaches. Treatment failure and disease relapse are attributed to extensive cellular and genetic heterogeneity existing not only between patients but also within a single tumor through space and time (10–13). This cellular heterogeneity, which is associated with clonal evolution, can be explained by the existence of multiple cellular subpopulations of cancer cells, called brain tumor initiating cells (BTICs), which have acquired stem cell properties including self-renewal, proliferation and multi-lineage differentiation capacity (14–17). Increased presence of chemo- (18) and radio-resistant (19) BTICs (14) plays a significant role in development of GBM treatment resistance and eventually tumor recurrence. Therefore, development of novel therapeutic modalities targeting BTIC populations is desperately needed for the GBM field.

Components of the tumor microenvironment play key roles in BTIC maintenance. A dominant microenvironmental factor of solid tumors including GBM is hypoxia (20). Intratumoral hypoxia has a significant effect on BTIC maintenance by supporting critical stem cell features including self-renewal, multipotency, tumorigenicity, and response to radiation (21, 22). In addition, hypoxia promotes cancer progression by inducing angiogenesis, cell growth, tumor cell invasion, genomic instability, immunomodulation, and metabolic reprogramming of cancer cells and tumor stroma (22–26). Therefore, targeting the hypoxic niche would be a necessary step towards decreasing BTIC survival and therapy resistance in GBM patients.

One of the most highly expressed genes in response to hypoxia is Carbonic Anhydrase 9 (CA9). CA9 is a cell surface metalloenzyme which catalyzes the reversible hydration of CO_2_ to produce protons (H^+^) and bicarbonate (HCO3^-^), permitting tumor cells to survive exposure to acidosis (25, 27–29). CA9 is highly overexpressed in response to hypoxia in many types of solid tumors including GBM (29–31). Furthermore, CA9 is also highly expressed in a large proportion of clear cell Renal Cell Carcinoma (ccRCC) (32) in a hypoxia-independent manner where it is driven by stabilization and constitutive activation of HIF-1alpha signaling as a result of mutation of the von Hippel-Lindau (VHL) tumor suppressor (33, 34). Clear cell Renal Cell Carcinoma is the most common form of renal cancer and like GBM is in need of improved therapeutic strategies. Patients with localized disease at diagnosis have a 5 year survival of 76%; however 30% of patients will present with metastatic disease which carries a dismal 5 year survival rate of 8% (35). Notably, CA9 displays limited expression in most normal tissues with the exception of gastrointestinal tract, gallbladder, and pancreatic ducts (36, 37). It has been shown that elevated CA9 expression is positively correlated with poor patient prognosis in a number of solid malignancies (29). The major therapeutic thrust targeting CA9 in solid tumors to date has been focused on small molecule inhibitors (38–40) and monoclonal antibodies such as G250 (41–43). While therapeutic benefit from single agent treatment is often quickly met with resistance (38, 44, 45), this has prompted investigation of combinatorial approaches with chemo- (38, 44, 45) and immunotherapy (46) which have enhanced therapeutic responses and expanded the possibility of therapeutic strategies to better target CA9 (47).

Here, we designed a T cell-based therapy that employs a Dual Antigen T-cell engager (DATE) antibody as a promising alternate strategy, which allows for targeting cancer cells and redirecting immune cells against tumor cells simultaneously. CA9 DATE was engineered by fusing the light chain of the CA9-Fab to OKT3, a single-chain fragment variable (scFv) construct that binds to the antigen-binding region of the mitogenic antiCD3ϵ clone. Bifunctional T cell engagers exhibiting specificity for the GBM tumor cell surface antigen CD133 (48)/EGFRvIII (49, 50) have also been shown to induce anti-tumorigenic activity in xenograft tumor models. Furthermore, we have previously demonstrated the utility of DATEs in eliminating GBM BTICs by targeting CD133 (51). Given their low molecular weight, DATEs may prove to be more efficient in localizing to the central nervous system (CNS) and this particular feature allows for maximal membrane proximity between the T cell and the cancer cell necessary for the immune response (52–55). However, DATEs are yet to be clinically translated for many solid tumors such as GBM and ccRCC.

We capitalized on the cell surface expression of CA9 and developed a DATE targeting its expression in solid tumors. To demonstrate proof-of-concept of our DATE in a model that highly expresses CA9 in a constitutive manner we utilized VHL mutant ccRCC PDX models. We demonstrate that the DATE is exquisitely specific for CA9 expressing patient-derived models of ccRCC and GBM. Simultaneous engagement of T cells and CA9^+^ target cells led to increased activation of T cells, increased inflammatory cytokine production and increased target cell death. Treatment of patient-derived models of ccRCC and GBM *in vivo* significantly reduced tumor burden and extended survival. This technology represents a new therapeutic strategy for hard-to-treat cancers highly expressing CA9.

## Material and Methods

### Human GBM and ccRCC Sample Collection

Human GBM brain tumors (**Table S1**) and patient-derived ccRCC cell lines (**Table S2**) were obtained from consenting patients, as approved by the Hamilton Health Sciences/McMaster Health Sciences Research Ethics Board and the Princess Margaret Cancer Centre, Toronto, respectively. Moreover, normal brain cells including Neural Stem Cells (NSCs) were isolated and propagated from fetal brain samples which was approved by the Hamilton Health Sciences/McMaster Health Sciences. Normal Human Astrocytes (NHAs) were purchased from Lonza.

### *In Silico* Analysis

Publically available databases including GEPIA2 and GlioVis were used for *in silico* validation of the target of interest across a large number of GBM samples.

### Culture Conditions for Isolating and Propagating the GBM and ccRCC Tumor Cells

Human brain tumor tissue was processed as previously described (14, 16, 56). Briefly, samples were dissociated in PBS (ThermoFisher, Cat#10010049) containing 0.2 Wünsch unit/mL Liberase Blendzyme 3 (Millipore Sigma, Cat#5401119001) and incubated on a shaker at 37°C for 15 minutes. The dissociated tissue was then filtered through a 70 μm cell strainer (Falcon, Cat#08-771-2) and collected by centrifugation at 1200 rpm for 5 minutes. Red blood cells were lysed using ammonium chloride solution (STEMCELL Technologies, Cat#07850). GBM cells were resuspended in NeuroCult complete (NCC) media, a chemically defined serum-free neural stem cell medium (STEMCELL Technologies, Cat#05751), supplemented with human recombinant epidermal growth factor (hrEGF) (20ng/mL: STEMCELL Technologies, Cat#78006), basic fibroblast growth factor (bFGF) (10ng/mL; STEMCELL Technologies Cat#78006), heparin (2 mg/mL 0.2% Heparin Sodium Salt in PBS; STEMCELL technologies, Cat#07980), antibiotic-antimycotic (1X; Wisent, Cat# 450-115-EL), and plated on ultra-low attachment plates (Corning, Cat#431110) and cultured as neurospheres. GBM BTICs Neurospheres were propagated by minimally-culturing (< 20 passages) human GBM samples and plating them on polyornithine- laminin coated plates for adherent growth. Adherent cells were replated in low-binding plates and cultured as tumorspheres, which were maintained as spheres upon serial passaging *in vitro*. These cells retained their self-renewal potential and were capable of *in vivo* tumor formation.

The human ccRCC cell lines were generated by sorting CA9-positive cells from patient tumor specimens as previously described (57). The ccRCC cell lines and their derived overexpression or knockout cell lines were grown in Iscove’s Modified Dulbecco’s Medium (IMDM) (ThermoFisher, Cat#12440053) supplemented with 10% fetal bovine serum (FBS, Thermo Fisher, Waltham) and 1% penicillin/streptomycin (ThermoFisher, Cat#15140122) at 37°C in 5% CO_2_. The murine cortical adenocarcinoma renal cell carcinoma cell line, Renca, was purchased from the American Type Culture Collection (ATCC). Renca and its derived overexpression cell lines were grown in Roswell Park Memorial Institute medium (RPMI 1640) (ThermoFisher, Cat#11875101) supplemented with 10% FBS and 1% penicillin/streptomycin at 37°C in 5% CO2.

### Generation of CA9 Knockout (KO) and CA9 Over-Expressed ccRCC Cell Lines

To perturb the carbonic anhydrase IX (CA9) gene in RCC243 for the generation of CA9-KO cell line, early-passage cells were transduced with lentivirus carrying Cas9 and guide RNAs (gRNA) targeting CA9 exons. The lentiviral expression vector lentiCRISPR v.2 (lcv2, Addgene) was purchased and modified in-house for gRNA compatibility. Three CA9-targeting gRNAs were individually cloned into the lcv2 vectors for lenti-virus production and transduction of RCC243 cells. The editing efficiency of the three gRNAs were verified using flow cytometry assessing surface CA9. The final CA9 knockout cells (RCC243 CA9-KO, gRNA sequence GGGATCAACAGAGGGAGCCA) were selected by fluorescence-activated cell sorting (FACS). To over express human CA9 in Renca (Renca hCA9), the mRNA open reading frame (ORF) was amplified from the human ORF transfection library (Dharmacon) using polymerase chain reaction (PCR), and Gibson-assembled into a lentiviral expression vector designed in-house. The CA9 ORF is linked to an enhanced green fluorescence protein (EGFP) *via* a P2A peptide. Early passage Renca cells were transduced with the CA9-lentivirus and FACS sorted based on positive EGFP signals. The surface CA9 levels of the validation cell lines were profiled by flow cytometry using phycoerithrin (PE)-conjugated anti-CA9 antibody (R&D systems, Cat# FAB2188P)

### Engineering and Production of CA9 DATEs

The complementarity-determining region (CDR) sequences of previously selected CA9-binders (generated by Dr. Sunandan Banerjee) were sub-cloned into the pSCSTa antibody expression vectors designed in-house, containing the OKT3-anti-CD3 single-chain variable fragment (scFv). The CDR-containing light and heavy chain variable regions of the F library phage-mids were amplified using PCR and restriction enzyme-digested to ligate with the pSCSTa backbone vectors. Both light- and heavy-chain pSCSTa expression vectors were then transfected into Expi293™ cells using the PEIpro^®^ transfection reagents (Polyplus, New York) following the manufacturer’s instruction. The transfected Expi293™ cells were cultured in the Expi293™ expression medium (Thermo Fisher, Waltham) and incubated at 37°C, 5% CO_2_ on a shaker. Five days post-transfection, the supernatant was harvested, and the antibody products were extracted by incubating with protein A resin and purified by affinity chromatography (Bio-Rad Laboratories, Hercules). The antibodies were exchanged into PBS buffer using Amicon^®^ Pro Purification tubes. The protein concentrations were measured using NanoDrop. The protein purity was verified using SDS-PAGE followed by Coomassie Blue staining.

### Flow Cytometry Analysis

GBM Tumorspheres were dissociated using 0.2 Wünsch unit/mL Liberase Blendzyme 3 (Millipore Sigma, Cat#5401119001) plus 10 μL DNase (Worthington Biochemical, Cat#LK003170) and adherent cultures were dissociated using dissociation enzyme TrypLE (ThermoFisher, Cat#12605028). The single cells were resuspended in PBS+2 mM EDTA (Invitrogen, Cat# AM9260G). Cells were then stained with APC conjugated mouse monoclonal human Carbonic Anhydrase 9 antibody (1:10) (R&D, Cat#FAB2188A) or a matched isotype control and CA9 DATEs followed by goat anti human APC-Fab IgG (1:2000, Jackson ImmunoResearch, Cat#109-136-170) and incubated for 15 minutes at room temperature. T cells were stained with CA9 DATEs (15 minutes RT) followed by goat anti human APC-Fab IgG (1:2000, Jackson ImmunoResearch, Cat#109-136-170), anti-CD25 (Miltenyi Biotech, Cat#130-113-283) and anti-CD69 (BD Bioscienecs, Cat#555533). Samples were run on a MoFlo XDP Cell Sorter (Beckman Coulter). Dead cells were excluded using the viability dye 7AAD (1:10; Beckman Coulter, Cat#A07704). Compensation was performed using mouse IgG CompBeads (BD Biosciences, Cat#552843). Samples were run on a MoFlo XDP Cell Sorter (Beckman Coulter) to assess the level of CA9 surface expression.

### Secondary Sphere Formation Assay (Self-Renewal Assay)

Tumorspheres were dissociated using 10 μL Liberase Blendzyme3 (0.2 Wunsch unit/mL) plus 10 μL of DNase in 1 mL PBS for 5 minutes at 37°C and adherent cultures were dissociated using dissociation enzyme TrypLE. CA9^+^ and CA9^-^ sorted GBM BTICs were plated at 200 cells per well in 200 μL of NCC media in a 96-well plate. Cultures were left undisturbed at 37°C, 5% CO_2_. The number of secondary spheres per well was counted at day 3 to 7 every day and used to estimate the mean number of spheres per 2,000 cells.

### Cell Proliferation Assay

Upon tumor culture dissociation, single cells were sorted into CA9^+^ and CA9^-^ population and 1,000 single cells were plated in 180 μL NCC per well in quadruplicate in a 96-well plate and incubated for five days. 20 microliters of Presto Blue (ThermoFisher, Cat#A13262), a fluorescent cell viability (metabolism) indicator, was added to each well approximately 4 hours prior to the readout time point. Fluorescence was measured using a FLUOstar Omega Fluorescence 556 Microplate reader (BMG LABTECH) at excitation and emission wavelengths of 544 nm and 590 nm, respectively. Readings were analyzed using Omega analysis software.

### Cell Growth in Hypoxic Condition

CA9^lo^ expressing GBM BTICs were cultured in both hypoxic and normoxic conditions. In hypoxic condition, cells were incubated in hypoxia chamber (1% O_2,_ 5% CO_2_, 94% N_2_) and in normoxic condition they were incubated in normoxia (21% O_2_) for a total of 5 days. After 5 days cultures were dissociated, and single cells were resuspended in PBS + 2 mM EDTA. Cells were then stained with mouse monoclonal human Carbonic Anhydrase 9 antibody (1:10) (R&D, Cat#FAB2188A) and run on the LSRII flow cytometer (BD) to assess the effect of hypoxia on CA9 expression on GBM BTICs.

### PBMC Isolation and T Cell Purification and Expansion

Peripheral blood mononuclear cells (PBMCs) from consenting healthy blood donors were obtained using SepMate ™ (STEMCELL technologies, Cat#85450) or Ficoll-Paque PLUS (GE Healthcare). This study was approved by the McMaster Health Sciences Research and the University of Toronto Ethics Board for GBM and ccRCC projects, respectively. 1 × 10^5^ cells in XSFM media (Irvine Scientific, Cat#91141) were activated with anti-CD3/CD28 beads at a 1:1 ratio (Dynabeads, Life Technologies) in a 96-well round bottom plate with 100U/mL rhIL-2 (Peprotech, Cat#200-02). T cell cultures were expanded into fresh media (XSFM media supplemented with 100U/mL rhIL-2) as required for a period of 12–15 days prior to experimentation.

### Binding Assay

The specificity of CA9 DATE for GBM cells, ccRCC cells and T cells were tested using flow cytometry analysis. CA9^hi^ GBMs, CA9^-^ GBMs, ccRCCs and T cells (isolated from human PBMCs for GBM study and Jurkat cells for ccRCC study) were resuspended in PBS plus 2 mM EDTA and were stained with CA9 DATEs followed by the secondary antibody, goat anti -human APC-Fab IgG (1:2000, Jackson ImmunoResearch, Cat#109-136-170) staining. GBM and ccRCC cells were incubated for 15 minutes at room temperature and for 20 minutes on ice, respectively followed by 15 minutes incubation at room temperature for the secondary antibody staining. Dead cells were excluded using the viability dye 7AAD (1:10; Beckman Coulter, Cat#A07704) and samples were run on MoFlo XDP Cell Sorter (Beckman Coulter) to assess the level of CA9 DATE binding to each of the above-mentioned lines.

### T Cell Activation Assays

In ccRCC model, RCC243 and RCC243 CA9-KO cells were plated at 200,000 cells/well in 6 well plates the night prior to treatment. Human CD3^+^ T cells at an E:T ratio of 5:1 were added to the wells along with (1 nM) or without CA9 DATEs and incubated at 37°C in 5% CO_2_ for 48 hours. The T cells were collected and stained for BV785 anti-human CD3 (BioLegend, Cat#317330), BV605 anti-human CD4 (BioLegend, Cat#317438), PE-anti-human CD8 (BioLegend, Cat#300908), and PE-CF594-anti-CD25 (BD Biosciences, Cat#562403) antibodies. Supernatants were collected and stored at -80°C for cytokine release analysis by enzyme-linked immunosorbent assay (ELISA).

In GBM models, GBM cells and T cells were co-incubated at a 1:1 ratio for 24 hours with (1μg = 13 nM) or without CA9 DATEs. The CD3^+^ (BD Pharmingen, Cat#563423) T cells and subpopulation of T cells including CD4^+^ (BD Pharmingen, Cat#555347) and CD8^+^ T (BD Horizon, Cat#562428) cells were analyzed for activation markers CD25 (Miltenyi Biotech, Cat#130-113-283) and CD69 (BD Pharmingen, Cat#555533) by flow cytometry. Supernatants were collected and stored at -80°C for cytokine release analysis by enzyme-linked immunosorbent assay (ELISA).

### Enzyme Linked Immunosorbent Assay (ELISA)

The concentration of TNF-α and IFN-γ were quantitated in the supernatant collected from the T cell activation assay (the T cell and GBM co-culture +/- CA9 DATE) using commercially available human TNF-α DuoSet ELISA kit (R & D Systems, Cat#DY210-05) and IFN-γ DuoSet ELISA kit (R & D Systems, Cat#DY285B-05) respectively. The sensitivity limits of TNF-α and IFN-γ assay were 15.60 pg/ml and 9.38 pg/ml, respectively. The experiment was performed in duplicates and the OD was measured at 450 nm using the FLUOstar Omega Fluorescence 556 Microplate reader (BMG LABTECH). The IFN-γ concentration in ccRCC model was quantified using the eBioscience Ready-SET-Go human IFN-γ ELISA kit (ThermoFisher, Cat#88-7386-88).

### Cytotoxicity Assay

To quantify target cell death without interfering signals from the effector cells, luciferase was overexpressed in ccRCC and GBM cells using lentiviral transduction (plasmidlenti-PGK-luciferase-GFP (Ailles Lab) for ccRCC cells and pCCL ffLuciferase for GBM cells).

ccRCC model: The RCC243 and RCC243 CA9-KO target cells were plated at 25,000 cells/well in triplicates in 96 well plate the night prior to treatment. The next morning, purified human CD3^+^ T cells were added at an E:T ratio of 5:1, along with CA9 DATE, and anti-CD3/BCMA control antibody at 1 nM concentration in standard complete IMDM media supplemented with 100 μM beta-mercaptoethanol (Sigma-Aldrich, Cat# M3148-25ML) to reduce T cell oxidative stress. The assays were incubated for 40-48 hours at 37°C, 5% CO_2_ before the microscopic images were documented and bioluminescence signals were measured. Working concentration (7.5 mg/mL) of firefly luciferin was added to the washed wells and read immediately with a spectrophotometer (BioTek plate reader).

Lactate dehydrogenase (LDH) release assays: Target cells (patient-derived ccRCC cell) were plated at 20,000 cells/well in 96 well plates (Corning, Cat#: 3628). Total PBMCs were used as effectors at an E:T ratio of 10:1. The LDH release by the damaged target cells were measured 48 hours later using the LDH cytotoxicity assay kit (Thermo Fisher, Cat# 88953). Prior to measuring, kit lysis buffer was added to three untreated wells of each cell line for the maximum LDH release controls. The assay steps were carried out as per manufacturer’s instructions. Briefly, supernatant from each treatment conditions was incubated with the LDH substrate mix at room temperature in dark for 20 minutes for the colour to develop. The stop solution was added and the plates were read using a spectrophotometer at 490 and 680 nm. The absolute LDH release was calculated as sample release, effector spontaneous release, target spontaneous release and medium blank. Relative LDH levels was calculated as sample absolute release/plus-PBMC-only absolute release.

GBM model: Luciferase-expressing GBM cells (CA9^hi^ GBM cells) and HEK cells at a concentration of 25,000 cells/well were plated in 96–well plates in triplicates. T cells at different effector-to-target (E:T) ratios (0:1, 0:0.25, 0.5:1, 0.75:1, 1:1, 2:1) were added to each well in the presence (1nM) and absence of CA9 DATE. The cultures were then incubated at 37°C for 18 hours. The next day 150 μg/mL firefly D-luciferin potassium salt (R&D systems, Cat#800-LN-05M) was added to each well and the BLI was measured with a luminometer (Omega) as relative luminescence units (RLU). Target cells incubated without effector cells were used to measure spontaneous death RLU. The readings from triplicates were averaged and percent lysis was calculated with the following equation:


% Specific lysis =100X (spontaneous death RLU – test RLU)/(spontaneous death RLU – maximal killing RLU)

### Animal Studies

Animal studies were performed according to guidelines under Animal Use Protocols (19-01-01) of McMaster University Central Animal Facility.

In ccRCC model, 5 x 10^6^ RCC243 VHL mutant cells were subcutaneously injected into the right flank of 8- to 10- week old immunocompromised NSG mice for tumor formation. After the half-maximal tumor engraftment (4 weeks post-engraftment) mice were randomly assigned into control or treatment groups based on matched-tumor size and CA9 DATE treatment started as described in **Supplementary Figure 2.** All animals received 12 doses of therapy within a 6-week time frame. The animals which were assigned to the treatment group were intratumorally injected with 50 μg CA9 DATE + 2 x10^6^ T cells (isolated from the freshly thawed PBMC of healthy donors) once a week and only 50 μg (667 nM) CA9 DATE for the second treatment in the week. Mice in the control group received the same therapy regimen as treatment group; however, the CA9 DATE was replaced with CA9 DATE control. Tumor size was measured using a ruler caliper after each treatment (length x width). To study the effect of CA9 DATE treatment on tumor size, tumors were collected one week after the last treatment and the tumor size was measured using a ruler caliper. For survival studies, all the mice were kept until they reached endpoint and the number of days for survival were noted for Kaplan-Meier analysis. The endpoint criteria were defined as 20% weight loss and 1.0 cm x 1.0 cm tumor size.

In GBM model, GBM cells (1x10^6^ BT935 and 2x10^5^ BT241) were intracranially injected into right frontal lobes of 6- to 8- week old immunocompromised NSG mice (bred in McMaster University Central Animal Facility) for tumor formation as previously described (58). Briefly, mice were anaesthetized using 2.5% Isoflurane (gas anaesthesia). Using a 15-blade scalpel a 1.0 cm vertical midline incision was made on top of the skull. A small burr hole was then made (2-3 mm anterior to the coronal suture, 3 mm lateral to midline) using a drill held perpendicular to the skull. A Hamilton syringe (Hamilton, Cat#7635-01) was used to inject 10 μL of cell suspension (GBM cells suspended in 10 mL PBS) into the frontal lobe. The syringe was inserted through the burr hole to a 5 mm depth. The incision was closed using interrupted stitches and sutures were sealed with a tissue adhesive. After the half-maximal tumor engraftment which was confirmed by MRI imaging (6 weeks post-surgery for BT935 and 10 days post-surgery for BT241) mice were randomly assigned into control or treatment groups and CA9 DATE treatment started as described in **Supplementary Figure 2**. All animals received 4 doses of therapy within a 2-week time frame. The animals which were assigned to the treatment group were intracranially injected with 50 μg (667 nM) CA9 DATE + 10^6^ T cells and received only 50 μg (667 nM) CA9 DATE top up each once a week for 2 weeks. Mice in the control group received the same therapy regimen as treatment group; however, the CA9 DATE was replaced with CA9 DATE control. For tumor volume evaluation, animals were perfused with 10% formalin one week after the last treatment and the collected brains were sliced at 2 mm thickness using brain-slicing matrix for paraffin embedding and H&E staining. Images were captured using an Aperio Slide Scanner and analyzed using ImageScope v11.1.2.760 (Aperio) and imageJ software. For survival studies, all the mice were kept until they reached endpoint and number of days of survival were noted for Kaplan-Meier analysis. The endpoint criteria were defined as 20% body weight reduction, physical appearance deterioration, measurable clinical signs, unprovoked behavior and response to external stimuli.

### Statistical Analysis

Biological replicates from at least three patient samples were compiled for each experiment, unless otherwise specified in figure legends. Respective data represent mean ± SEM, n values are listed in figure legends. Cox regression and Kaplan-Meier analysis were performed for survival analysis. Student’s t-test analyses, 2-way ANOVA analysis and Kaplan-Meier analysis were performed using GraphPad Prism 6. P<0.05 was considered significant.

### Study Approval

This study was conducted with approval from the Hamilton Integrated Research Ethics Board for human studies and the Animal Research Ethics Board at McMaster University.

## Results

### *CA9* Is a Safe Target for GBM Immunotherapy

**Figure 1:**
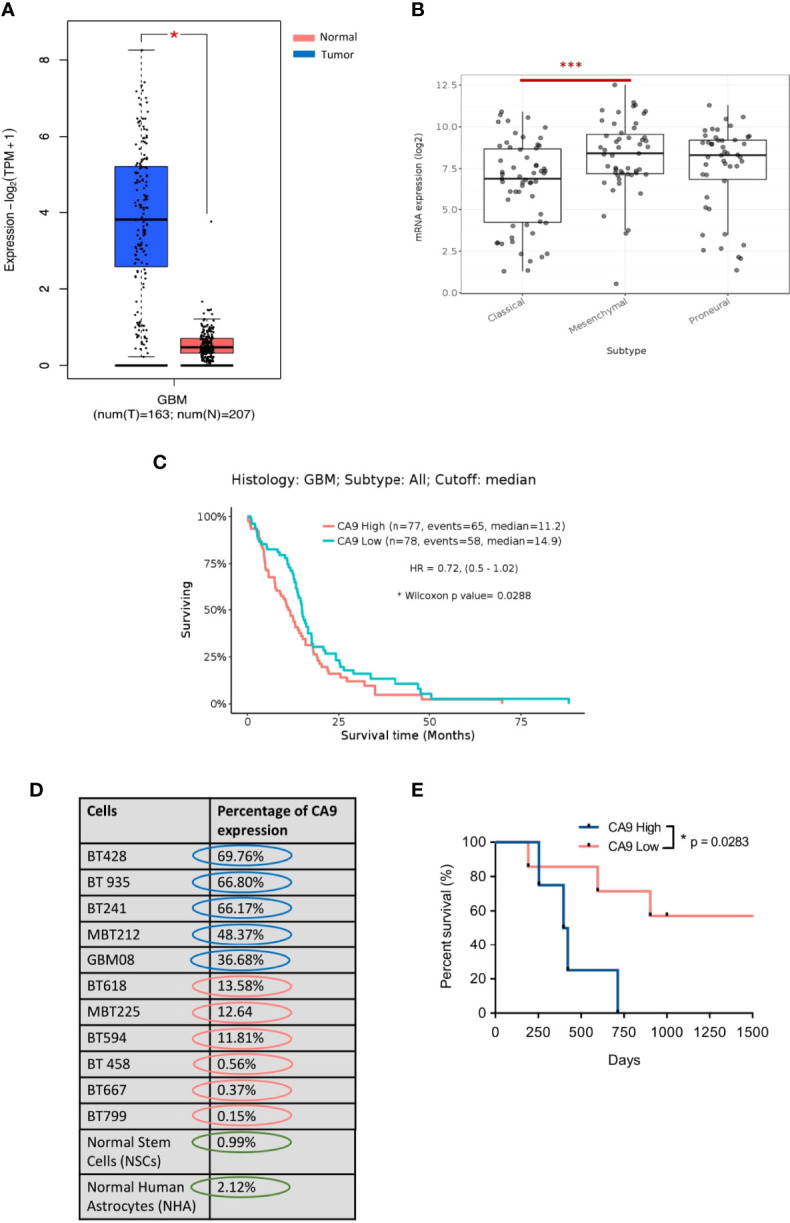
CA9 as a therapeutic target in GBM. **(A)** Transcriptomic dataset shows significant upregulation of CA9 in GBM samples (n=163) when compared to non-tumor (n=207) (GEPIA2) (P value: * < 0.05). **(B)** CA9 has higher expression in mesenchymal (Mes) subtype (n=51) compared to proneural (PN) (n=46) and classical (Cla) (n=59) of GBM (GlioVisTCGA) (P value:***< 0.001). **(C)** Survival data from the TCGA dataset for *CA9* high (n=77) transcript expression of GBM samples illustrating a significant increase in survival when compared to *CA9* low (n=78) samples (Median *CA9* mRNA expression (log2) cut off: 7.66; HR: 0.72 (0.5-1.02); Wilcoxon p value: 0.0288). **(D)** Characterisation of surface CA9 expression of GBM samples along with normal stem cells (NSCs) and normal human astrocytes (NHAs) in normoxic condition by flow cytometry reveals varying expression of CA9 in GBM lines, but low levels in normal cells. **(E)** GBM samples (n=11) from Fig 1.D. were grouped into either CA9low (red, n=5) or CA9high (blue, n=4) expression based on a flow cytometric median of 20%. Log-rank (Mantel-Cox Test) analysis demonstrated a significant survival benefit for patients bearing CA9Iow tumors with a median survival of 33 and 13.5 months for patients bearing CA9Iow and patients bearing CA9high tumors, respectively (P value: * =0.0283).

To evaluate the potential utility of CA9 as a therapeutic target in GBM, we performed *in silico* analysis using the GEPIA2 database to assess the level of *CA9* expression in GBM versus normal tissue. This analysis revealed a significant upregulation of *CA9* in GBM tissue compared to normal tissues (**Figure 1A**). We next evaluated CA9 expression in the TCGA glioma database and identified higher expression of *CA9* in GBM (grade IV glioma) compared to low-grade glioma (**Figure S1A**). Notably, this *in silico* analysis revealed significantly higher expression of *CA9* in the mesenchymal subtype, the most aggressive GBM subtype (**Figure 1B**). Furthermore, *CA9* expression was also significantly higher in all subtypes of GBM compared to normal tissue (**Figure S1B**). Stratifying patients according to median *CA9* expression revealed patients with increased *CA9* expression survived for shorter periods (59, 60) (**Figure 1C**). We next validated our *in silico* findings at the protein level using flow cytometry analysis to evaluate cell surface CA9 expression in a cohort of our patient-derived GBM BTIC lines as well as in normal brain cell lines [Normal Human Astrocyte (NHA) and Neural Stem Cells (NSC)]. We identified very little CA9 expression on normal brain cells (NHAs and NSCs), and strong extracellular/cell surface expression in our patient-derived GBM BTIC lines (**Figure 1D**). Since this respository is linked to patient outcome data we next investigated whether CA9 expression impacted the survival of the patients from which these lines were derived. Our analysis revealed a positive correlation between CA9 expression and poor patient survival (**Figure 1E** and **Table S1**) suggesting that our findings at the mRNA level are extended to expression of the protein. Together, these data suggest that CA9 is an important therapeutic target in GBM and given its cell surface expression and limited expression in normal brain samples, immuno-therapeutic modalities targeting cells expressing CA9 have reduced risk of off-target toxicity in the brain.

### CA9 Influences BTIC Stem*-*Like Properties

**Figure 2:**
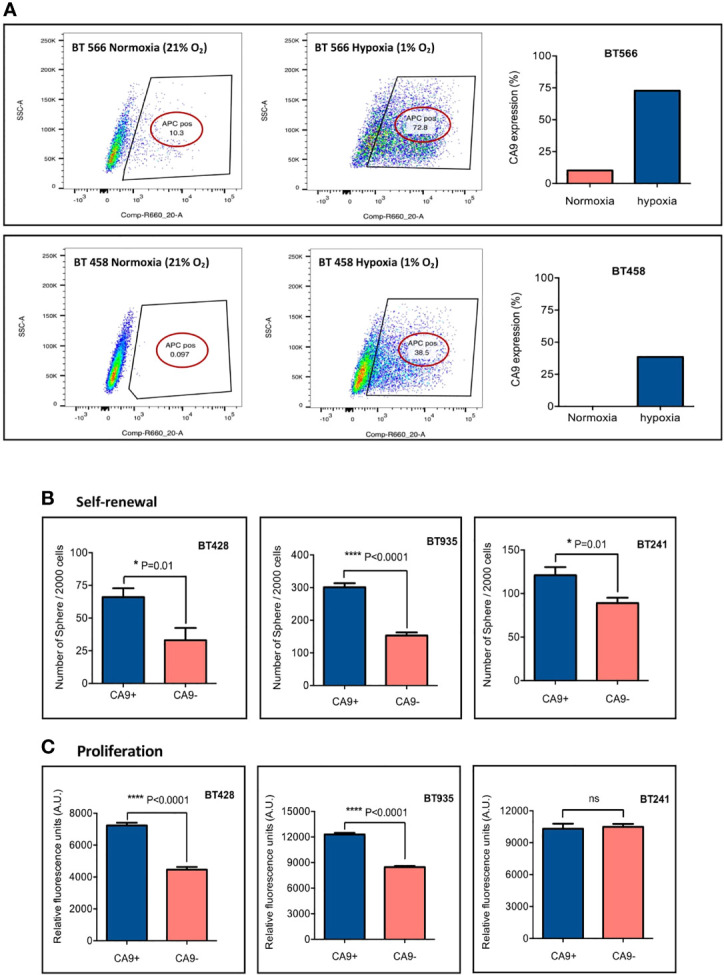
CA9 expression elevates in hypoxic condition and is associated with higher level of self-renewal and proliferation. **(A)** CA9 surface expression was evaluated after cells were cultured in normoxia or hypoxia (1% O_2_) for 5 days; and the results indicated a dramatic increase in CA9 expression on both GBM BTIC lines upon exposure to hypoxia. **(B)** Significant increase of self-renewal capacity as measured by secondary sphere formation assay and **(C)** proliferative potential as measured by PrestoBlue proliferation assay is seen in CA9+ when compared to CA9- cells. (P value: **** <~0.0001, * 0.01, ns: non-significant) (mean±SEM, two-tailed t-test).

To investigate the effect of hypoxia on CA9 expression in our patient-derived GBM BTIC lines, CA9^lo^ expressing GBM BTICs were cultured side by side in hypoxic (1% O_2_) and normoxic conditions. Cell surface characterization by flow cytometry indicated that cells cultured in hypoxic conditions had significant elevation of CA9 expression compared to cells which were cultured in normoxic conditions (**Figure 2A**).

There is accumulating evidence about the effect of the tumor microenvironment, particularly hypoxia, on BTIC maintenance and treatment resistance (61, 62). It has also been shown that hypoxia can induce a stem-like phenotype in non-stem-like cancer cells, promoting cell growth and self-renewal (62). Moreover, CA9 has previously been implicated in BTIC survival (38). To assess the influence of CA9 on GBM BTIC stem-like properties including self-renewal and proliferation, secondary sphere formation and proliferation assays were performed. Each GBM BTIC line was sorted into CA9^+^and CA9^-^ fractions by FACS and plated to assess self-renewal and proliferation. The expression of CA9 significantly enhanced clonogenicity of the BTIC lines (**Figure 2B**). Moreover, in 2 out of 3 GBM BTIC lines tested, the CA9^+^ cell population had significantly greater proliferative capacity than the CA9^-^ cell fractions (**Figure 2C**). Altogether, these data revealed that CA9 influences stem-like properties in GBM BTICs where targeting cells expressing CA9 may be beneficial.

### CA9 Dual Antigen T Cell Engager (DATE) Is Specific for CA9 and CD3

To target CA9 expressing tumor cells, we exploited the cell surface localization and generated DATEs. CA9-specific DATEs were engineered by fusing the antigen-binding portion (Fab) of the anti-CA9 antibody to the antigen-binding region of mitogenic anti-CD3ε clone (scFv OKT3) with a short flexible amino acid linker (63). This engineered CA9 DATE consists of a ~50 kDa CA9 Fab-light chain fused to OKT3 scFv and a ~25 kDa CA9 Fab-heavy chain.

**Figure 3:**
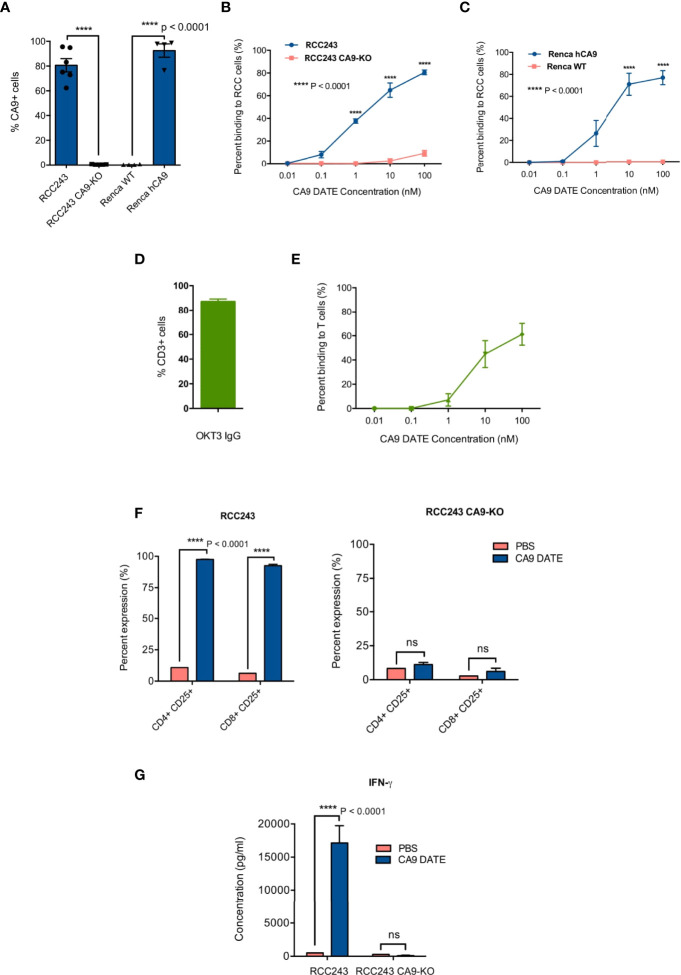
Generation and assessment of human anti-CA9 Dual specific T cell engagers (DATEs) on Renal Cell Carcinoma (ccRCC). **(A)** CA9 expression level on RCC243, RCC243 CA9-KO, Renca and Renca hCA9 cells. **(B)** Increasing concentrations of CA9 DATE binding to RCC243 vs RCC243 CA9-KO, **(C)** Renca hCA9 vs Renca WT cells and **(E)** CD3 expressing Jurkat cells were measured using flow cytometry. Error bars: mean ± SEM. **(D)** okt3 expression level on Jurkat cells. **(F)** Addition of CA9 DATEs (1 nM) to the co-culture of human CD3+ T cells with CA9 expressing target cells (Luciferase-expressing RCC 243 cell lines and the CA9 knockout counterpart [CA9-KO]) for 48 hours at the E:T ratio, 1:5 resulted in a significant elevation of CD25 expression on both CD4+ and eng+ T cells population confirmed by flow cytometry analysis. (n=4) (P value: **** < 0.0001) (2 way ANOVA) **(G)** Enzyme-linked immunosorbent assay (ELISA) indicated increased secretion of Interferon-gamma (IFN-y) by T cells only in the presence ofCA9 DATE and CA9 expression on target cells. (P value:**** < 0.0001) (2 way ANOVA).

We performed binding assays to test the dual specificity of the purified DATEs for CD3 on T cells and CA9 on patient-derived tumor cells. As a proof-of-principle we first assessed DATE specificity in patient-derived models of ccRCC where CA9 is constitutively expressed, irrespective of oxygen tension, due to *VHL* loss (RCC243) and in the murine cortical adenocarcinoma renal cell carcinoma cell line (Renca) where we overexpressed CA9. Endogenous, cell surface expression of CA9 was found in >80% of RCC243 cells which was completely lost upon CRISPR knockout of the gene (**Figure 3A**). Similarly, overexpression of human CA9 (hCA9) in Renca cells resulted in 90% of the cells positive for cell surface expression of CA9, which was undetectable in the wild type cells (**Figure 3A**). We stained the RCC243 and RCC243 CA9 KO as well as WT and hCA9 Renca cells with varying concentrations of the CA9 DATE. CA9 binding was only observed in CA9-expressing lines (RCC243, Renca hCA9) and no binding was detected in CA9-negative lines (RCC243 CA9 KO and Renca WT) demonstrating the specificity of the CA9 DATE to bind target cells in an antigen (CA9) restricted manner (**Figures 3B, C**). We next confirmed DATE binding to T cells using Jurkat cells which were 90% positive for CD3 expression (**Figure 3D**). Again, we observed a dose-dependent increase in DATE binding to Jurkat cells (**Figure 3E**). Together, our results confirmed antigen specificity and high affinity binding of CA9 DATEs to the tumor cells and T cells.

### CA9 DATE Activates T Cells and Induces Tumor Cell Lysis

The main mechanism of action for the CA9 DATE upon binding to T cells and tumor cells is T cell activation and subsequent tumor cell lysis. Upon confirming the antigen specificity of the CA9 DATE for both T cells and tumor cells, we aimed to assess the efficacy of the CA9 DATE by further investigating its ability to activate and re-direct human T-cells against antigen-expressing tumor cells. For this purpose, T cells were co-cultured with ccRCC cells in the presence and absence of DATEs. We evaluated T-cell activation by staining for CD25 and detected elevated expression of CD25 (late activation marker) in both CD4 and CD8 T cells only in the presence of CA9 DATEs (**Figure 3F**; left). Moreover, this effect was entirely dependent on the expression of CA9 by the target cells (**Figure 3F**; right), suggesting the DATE was permitting the formation of an immunological synapse between the T cells and ccRCC cells leading to T cell activation. Furthermore, T cell activation was further confirmed by the increase in IFN-γ production in the presence of the DATE in a CA9-dependent manner (**Figure 3G**). Together, these results demonstrated that the CA9 DATE has the ability to activate T cells in a strictly antigen-dependent manner.

**Figure 4:**
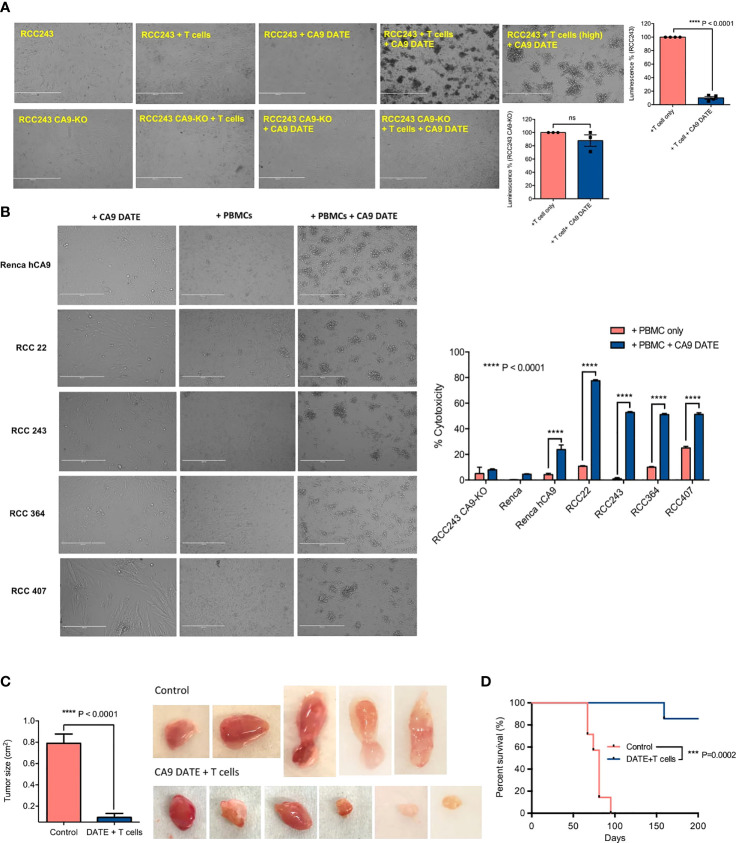
Assessing the efficacy of CA9 DATE in therapeutic targeting of CA9 expressing RCC lines and xenografted immunocompromised mice: **(A)** CA9 DATE (1 nM) induced potent cytolysis in antigen expressing target cells when cocultured with human CD3+ T cells at an E:T ratio of 5:1 for 48 hours quantified by luminescence assay. Phase contrast images of the *in vitro* cytotoxicity assay confirmed potent cytolytic effect of CA9 DATE on CA9 antigen expression. (n=4) (Scale bar: 1000 µ) (P value: ****< 0.0001, ns, non-significant) (mean SEM, two-tailed t-test). **(B)** CA9 DATE effectively induced target lysis across a panel of kidney cancer cell lines when co-cultured with CD3+ T cells at E:T ratio of 10:1 *(In vitro* cytotoxicity assay setup as described earlier). lactate dehydrogenase (LDH) release assay on the supernatant indicated a drastic increase in target cell cytotoxicity in the presence of CA9 DATE and CA9 antigen expression. The phase contrast images of light microscope confirmed the cytolytic effect of CA9 DATE on CA9 expressing RCC lines (n=2). (P value:****< 0.0001) (2 way ANOVA). **(C)** NSG mice were subcutaneously implanted with human CA9+ RCC 243 VHL mut cells. Upon successful engraftment and having a palpable tumor, mice were intratumorally treated with 2x10^6^ T cells isolated from human PBMCs either with CA9 DATE or CA9 DATE control (50µg) for a total of 12 doses over 6 weeks. Mouse xenografts generated after CA9 DATE treatment had less tumor burden (n=6) (P value: **** < 0.0001) (mean±SEM, two-tailed t-test) and **(D)** maintained a significant survival advantage over control mice (n=7) (P value: *** < 0.0004) (Log-rank Mantel-Cox Test).

We next assessed the efficacy and potency of CA9 DATE-directed T-cell cytotoxicity. Co-culture of RCC243 cells with T cells in the presence of the CA9 DATE resulted in significant cell death (**Figure 4A**). In contrast, no cell death was detected when RCC243 CA9 KO cells were cultured with T cells in the presence of the DATE (**Figure 4A**). We then extended our findings to 3 additional patient-derived models of ccRCC as well as the Renca-hCA9 model (**Figure 4B**). Co-culture of these lines with PBMCs resulted in significant target cell death only when the DATE was present (**Figure 4B**). Altogether, these data strongly confirmed that CA9 DATE can potently activate T cells and redirect them to tumor cells triggering robust tumor cell death.

### CA9 DATE Inhibits ccRCC Tumor Growth

To evaluate the effect of the CA9 DATE on tumor growth *in vivo* we evaluated the RCC243 model where CA9 expression is highly, homogeneously expressed throughout the tumor. RCC243 *VHL* mutant lines sorted for CA9 expression and CA9^+^ cells were injected into the flank of immunocompromised NSG mice. Upon tumor formation, mice were co-injected with DATEs and isolated T cells from freshly thawed human PBMCs intratumorally (**Schematic Figure S2A**). Mice treated with CA9 DATE and T cells had significantly reduced tumor growth (**Figure 4C**) which translated to a significant survival benefit (**Figure 4D**) over the control arm. Thus, these data provide excellent evidence that the CA9 DATE can be effectively utilized to treat CA9 expressing solid tumors independent of hypoxia.

### CA9 DATE Binds *CA9* on GBM BTICs, Activates T Cells and Induces Target Cell Death

**Figure 5:**
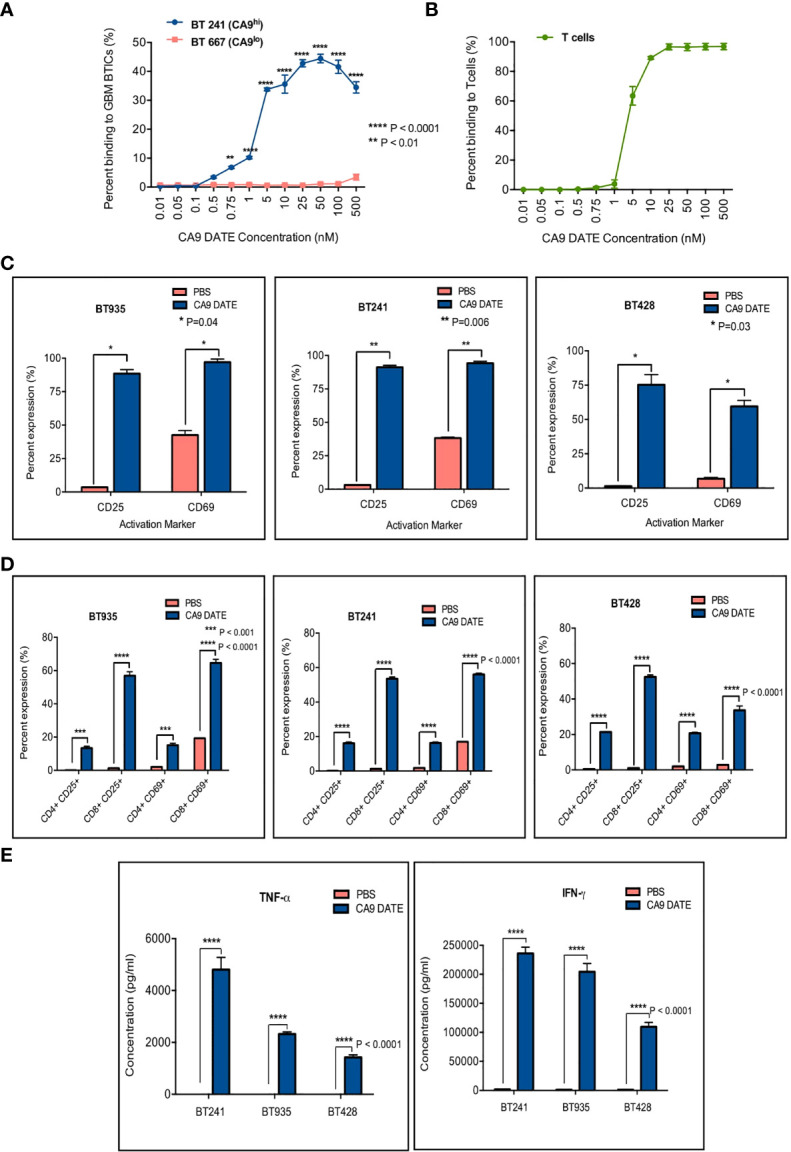
Assessment of anti-CA9 Dual specific T cell engagers (DATEs) dual specificity and its effect on GBM model. **(A)** Dual specificity of CA9 DATEs on CA9hi GBM BTIC (BT241), CA9low GBM BTIC (BT667) and **(B)** human PBMC derived T cells by flow cytometry. (P value: **<0.01, **** <0.0001) (2-way RM AVOVA). **(C)** Addition of CA9 DATE (1µg/mL) (13nM) to the co-culture of CA9hi GBM BTICs (BT241, BT935, BT428) and T cells (E:T ratio, 1:1) (overnight incubation) caused T cells activation as confirmed by increased expression of CD25 and CD69 by flow cytometry analysis (n=2). (P value: *= 0.04, **= 0.006) (2 way ANOVA) **(D)** CD8+ T cells were the main subset of activated T cells. (P value: ***< 0.001, **** < 0.0001) (2 way ANOVA) **(E)** Enzyme-linked immunosorbent assay (ELISA) shows elevated secretion of IFN-γ and TNF-α cytokines in supernatant collected from co-cultures ofT cells and GBM BTICs treated with CA9 DATEs. (n=2) (P value: **** < 0.0001).

We next sought to determine the efficacy of the CA9 DATE in GBM using our patient-derived GBM BTIC lines. We first performed binding assays on CA9^hi^ (BT241) and CA9^lo^ (BT667) GBM lines (**Figure 5A**) to determine the DATE binding capacity in these GBM cells. Similar to our ccRCC observations (**Figure 3B**), we observed a dose-dependent increase in DATE binding to BT241 cells that was not detected in the BT667 line (**Figure 5A**) confirming the antigen specificity of the DATE. We next confirmed the ability of the DATE to bind T cells derived from human PBMC (**Figure 5B**). The DATE bound T cells in a dose-dependent, saturable manner.

We next assessed whether the co-culture of T cells and GBM cells in the presence of the DATE would also lead to activation of the T cells. We stained T cells for CD25 and CD69 after an overnight co-culture with BT935, BT241 and BT428 with or without the CA9 DATE (**Figure 5C**). T cell activation was observed in all 3 co-cultures in a DATE-dependent manner. Moreover, this activation was greatest for CD8 T cells (**Figure 5D**). Furthermore, T-cell activation was also associated with the elevated secretion of pro-inflammatory cytokines TNFα and IFNγ in a DATE-dependent manner (**Figure 5E**).

**Figure 6:**
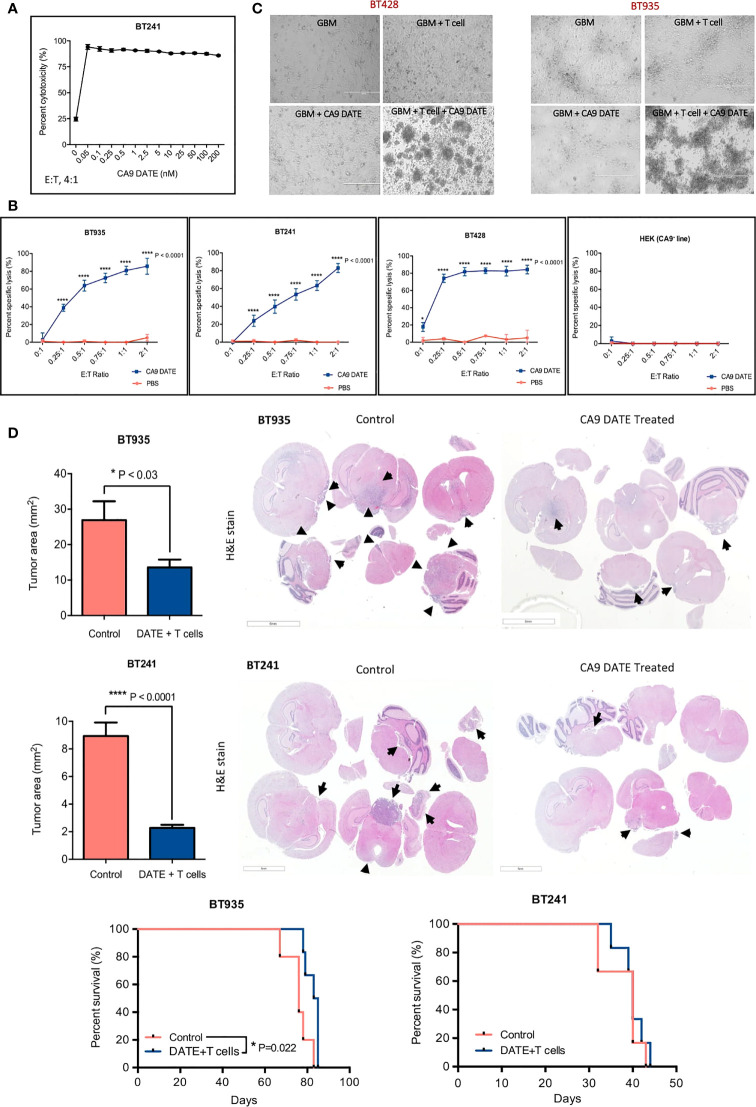
Therapeutic targeting of CA9hi GBM BTICs using CA9 DATEs at *in vitro* level and in patient derived xenograft model of GBM. **(A)** Dose response study performed on BT 241, CA9hi GBM BTIC, identified 1 nM as the optimal dose for GBM cytotoxicity assay. **(B)** DATEs significantly induced cytotoxicity of CA9hi GBM BTICs (BT241, BT935, BT428) but not CA9- cells (HEK) when co-cultured with T cells and DATEs (1n M) for 16 hours at different E:T ratios. (n=3) (P value:*< 0.05, **** < 0.0001) (2 way ANOVA). **(C)** Micrographs of GBM BTICs and T-cell co-culture with and without DATEs. CA9hi GBM BTIC lines (BT 935 and BT 428) were incubated with either T cells (E:T ratio, 1 :2) or CA9 DATE alone or with both. GBM BTIC lysis were observed only in the presence of both T cells and CA9 DATEs (Scale bar: 400 m). **(D)** NSG mice were intracranially implanted with human CA9hi GBM BTICs (BT935 and BT241). Upon successful engraftment, mice were intracranially treated with 1x10^6^ T cells isolated from human PBMCs either with CA9 DATE or CA9 DATE control (50µg = 667nM) for a total of four doses over two weeks. Mouse xenografts generated after CA9 DATE treatment had less tumor burden (n=6) (P value: * < 0.03, **** < 0.0001) (mean±SEM, two-tailed t-test) and maintained a significant survival advantage over control mice in BT935 engrafted mice (P value: * < 0.03) (Log-rank Mantel-Cox Test); however, DATE treatment on BT241 engrafted mice only showed increased pattern of survival (n=6).

We next sought to determine whether the enhanced T cell activation following exposure to the CA9 DATE in the presence of CA9 would lead to GBM lysis. To determine the concentration at which the CA9 DATE has optimal cytotoxicity on GBM BTICs, we titrated the DATE from 0 nM to 200 nM in co-cultures of GBM BTICs and T cells (**Figure 6A**). Remarkably, even the lowest concentration of CA9 DATE (50 pM) invoked a potent cytolytic effect on GBM BTICs (**Figure 6A**). Ultimately, 1 nM was chosen as the best concentration for performing cytotoxicity assays. We then evaluated cytotoxicity across multiple E:T ratios at a constant DATE concentration and identified significant cell death was induced as low as at a E:T ratio of 0.25:1 (**Figure 6B**). Furthermore, this was only observed in the presence of DATE and CA9. In addition, microscopic examination confirmed that co-incubation of T cells (suspension) and GBM BTICs (adherent) in the presence of CA9 DATEs leads to GBM BTIC lysis. In contrast to wells without DATEs, the co-cultures with CA9 DATEs showed detachment of target GBM cells that formed rosettes, indicating clumps of dying cells (**Figure 6C**).

### CA9 DATE Reduces GBM Tumor Growth and Extends Survival

We next assessed the efficacy of the CA9 DATE *in vivo* using two separate early passage patient-derived GBM cell lines enriched for BTIC populations (BT935 and BT241). BT935 cells were injected intracranially into immunocompromised NSG mice. Following engraftment, CA9 DATEs and isolated T cells from freshly thawed human PBMCs were co-injected intracranially (**Figure S2B**). Treatment with the CA9 DATE and T cells significantly reduced tumor growth upon completion of the treatment regimen, whereas tumor growth was unaffected in mice receiving the control DATE and T cells (**Figure 6D**). Furthermore, the CA9 DATE-T cell regimen led to an extension in mouse survival (**Figure 6D**). We then tested the efficacy of the DATE in a second orthotopic patient-derived GBM model. Following engraftment of BT241 tumors mice were treated with the CA9 DATE and T cell regimen (**Figure S2C**). Again, the CA9 DATE and T cell regimen led to a significant reduction in BT241 tumor growth upon completion of the treatment regimen. However, this failed to provide a survival benefit in this model (**Figure 6D**). In summary, we have developed a highly specific CA9 targeting DATE that potently activates T cells and effectively controls growth of CA9-positive solid tumors.

## Discussion

In the present study we developed a novel immunotherapeutic approach to target CA9 utilizing a CA9-specific DATE. The CA9 DATE was able to bind CA9 and redirect T cells to ccRCC and GBM cells in a CA9-dependent manner. Furthermore, the DATE possessed potent antitumor activity in multiple patient-derived solid tumor models of GBM and ccRCC containing both robust constitutive CA9 expression in response to *VHL* loss as well as in models with microenvironmentally-driven CA9 upregulation. While survival benefit from our studies was substantive in the ccRCC models, this was not the case in our GBM models. This is perhaps a reflection of the higher antigen levels present throughout the tumor at the initiation of dosing in the ccRCC models versus the GBM models together with the half-life of the administered DATE and the number of times it can be administered. It thus requires development of an optimized dosing regimen. Consequently, it is difficult to extrapolate these findings to the patient condition given that the dose and dosing regimen are likely to be entirely different in humans and patients will have undergone or will be undergoing standard of care treatment concurrently compared to our single agent experiments. Standard of care in GBM has left the dismal patient outcome associated with this disease unchanged over the last two decades (5). The tremendous degree of heterogeneity within GBM tumors combined with the influence of the tumor microenvironment has contributed significantly to this (64). GBMs are quite hypoxic (22, 65, 66) and BTICs are enriched in areas of hypoxia (22, 61, 62). Here we provide evidence that CA9 expression is important for BTIC self renewal and proliferation, suggesting that our CA9 DATE has the potential to eliminate BTIC cells within GBMs. These data align with previous findings demonstrating that combining a small molecule targeting CA9 activity with temozolomide reduces the GBM BTIC population (38). Since BTICs are a significant contributing factor to treatment resistance and disease recurrence (18, 19), our CA9 targeting DATE may be used to overcome treatment resistance and help prevent the recurrence of GBMs.

The development of novel immunotherapeutic strategies to engage the immune system to treat GBMs have provided renewed hope for improving patient outcomes (67, 68). GBMs are typically classified as an immunologically cold tumor (69) and contain T cells at low abundance (70, 71). Overcoming this with strategies that can direct the T cells to critical tumor associated antigens has led to the development of bifunctional T cell engagers which are currently being evaluated clinically in GBM (68). Here we propose that CA9 is an important tumor associated antigen that may be exploited using this strategy. Our data demonstrates long term efficacy of the DATE in tumors with CA9 expressed homogeneously throughout the tumor at a high level, yet short term efficacy in models where the proportion of CA9 positivity is lower. These findings demonstrate that the DATE is very effective at eliminating antigen positive cells and that recurrence is perhaps a reflection of both regrowth driven by antigen negative cells (68) and the short half-life of the DATE (72). This suggests that the CA9-DATE treatment strategy can benefit from combinatorial tumor targeting strategies such as CAR T therapies or oncolytic viruses, including those that are engineered to secrete bifunctional T cell engagers (73–75), targeting additional GBM tumor associated antigens along with CA9. Moreover, CA9 inhibition has been shown to enhance the efficacy of immune checkpoint inhibitors (ICIs) in hypoxic solid tumors (46). While this approach has yet to provide substantial benefit in GBM, combining the ICIs with the DATE and injected T cells may be worth further exploration in this setting where some of the immunosuppressive features of the GBM have been overcome by targeting CA9.

CA9 expression while highly upregulated in solid tumors, is also present minimally in normal tissues (36, 37) which may pose on-target, off-tumor toxicity challenges. This is precisely why CAR-T strategies targeting CA9 have failed (76). However, on-target-off-tumor toxicity appears to be less of a concern in antibody mediated therapy as monoclonal antibody targeting of CA9 has been used for imaging (77) and radio-immunotherapy (78, 79) without significant GI toxicity issues. Thus, persistence of CA9 targeting may be the culprit responsible for the biliary toxicity associated with CA9 CAR-T therapies, but not antibody mediated therapies. The fact that this strategy requires both the target antigen to be expressed (CA9) and the T cells to be in the same location likely adds safety to the approach and potentially mitigates the concerns about on-target-off-tumor toxicity. Furthermore, local delivery of the DATE will further aid in eliminating systemic on-target-off-tumor toxicity. Nevertheless, this is a concern that will be evaluated, along with delivery strategies, as this therapeutic approach progresses through preclinical and clinical testing.

In summary, our data strongly supports the utility of CA9 DATEs against GBM and ccRCC tumors highly expressing CA9. This antibody-based targeted therapy may expand our arsenal of effective therapeutic approaches targeting CA9 in solid tumors and warrants further exploration.

## Data Availability Statement

The original contributions presented in the study are included in the article/**Supplementary Material**. Further inquiries can be directed to the corresponding author.

## Ethics Statement

Human GBM brain tumors and patient-derived RCC cell lines were obtained from consenting patients, as approved by the Hamilton Health Sciences/McMaster Health Sciences Research Ethics Board and the Princess Margaret Cancer Centre, Toronto, respectively. The patients/participants provided their written informed consent to participate in this study. The animal studies were performed according to guidelines under Animal Use Protocols (19-01-01) of McMaster University Central Animal Facility.

## Author Contributions

NT, CV, XZ, JM, and SS conceived and designed overall studies. NT, DM, and NS performed animal studies. NT, XZ, MSh, and SC propagated brain tumor lines and performed *in vitro* assays. Flow studies were performed by MSu. NT, CV, XZ, and KL perfomed data interpretation. MSe helped with assessing the effect of hypoxia on target expression. NT developed and edited figures and performed statistical analyses. NT, SC, and CV drafted and edited the manuscript, with input from all authors. All authors contributed to the article and approved the submitted version.

## Funding

This work was supported through a program project grant from the Terry Fox Research Institute Canada (#1065) (to SS and JM) and funds to SS from McMaster University Department of Surgery. NT was supported by MITACS fellowship in partnership with Center for the Commercialization of Antibodies and Biologic.

## Conflict of Interest

The authors declare that the research was conducted in the absence of any commercial or financial relationships that could be construed as a potential conflict of interest.

## Publisher’s Note

All claims expressed in this article are solely those of the authors and do not necessarily represent those of their affiliated organizations, or those of the publisher, the editors and the reviewers. Any product that may be evaluated in this article, or claim that may be made by its manufacturer, is not guaranteed or endorsed by the publisher.
