# Supplementary material for: Dual Antigen T Cell Engagers Targeting CA9 as an Effective Immunotherapeutic Modality for Targeting CA9 in Solid Tumors
Source: Front Immunol. 2022 Jul 6;13:905768. doi: 10.3389/fimmu.2022.905768 (PMC9296860; doi:10.3389/fimmu.2022.905768)
Supplement: Supplementary file 1 [file DataSheet_1.pdf]

Supplementary Figure. 1

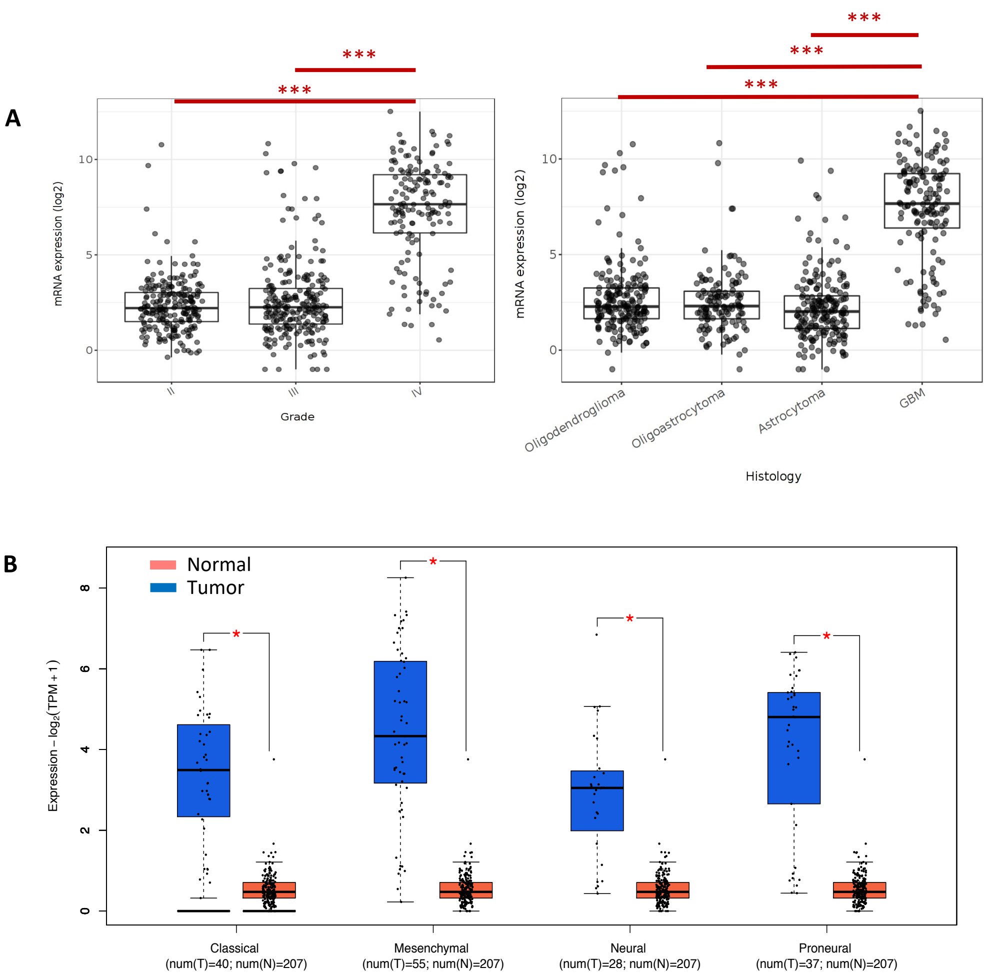

Supplementary Figure. 1. CA9 expression in glioblastoma. A) CA9 has higher expression in GBM (grade IV) (n=150) compared with low-grade gliomas (II and III) (n=226 and 244 respectively), oligodendroglioma (OG) (n=191), and astrocytoma (Astro) (n=194) at the transcriptomics level according to the GlioVis database (TGCA) (P value: *** < 0.001). **B)** CA9 expression is higher in each subtype compared to normal tissue (PEGIA2). (P value * < 0.05, ***, P < 0.001).

Supplementary Figure. 2

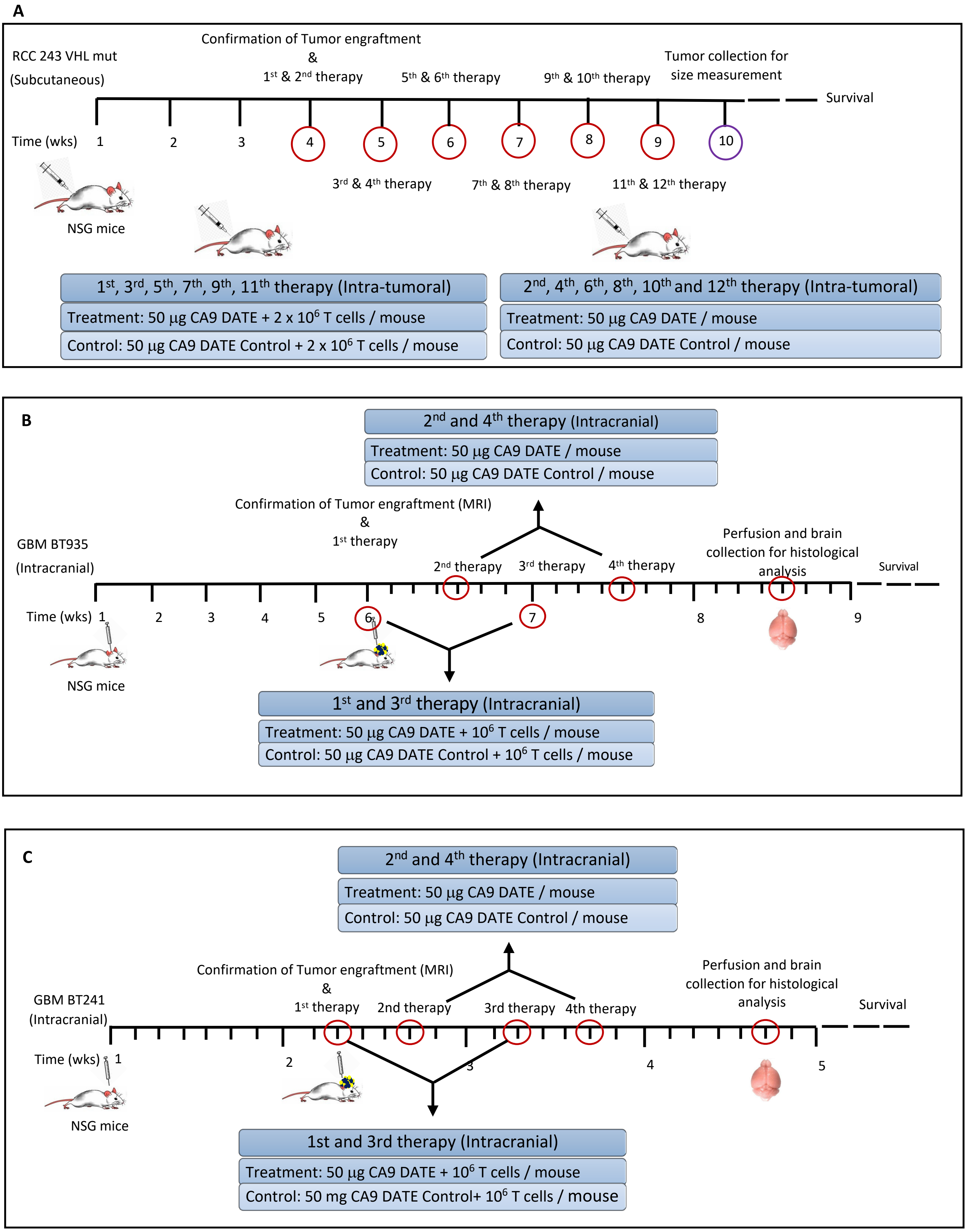

Supplementary Figure. 2. The schematic of *in vivo* preclinical testing of CA9xCD3 DATE antitumor activity using patient derived xenograft model: A) Subcutaneous implantation of human $CA9^{+}$ RCC 243 VHL mut cells for generating RCC model and the detailed treatment plan. **B** and **C)** Intracranial engraftment of human $CA9^{hi}$ GBM BTICs (BT935 and BT241) for generating GBM model and the detail of treatment regimen.
