# Supplementary material for: Dual Antigen T Cell Engagers Targeting CA9 as an Effective Immunotherapeutic Modality for Targeting CA9 in Solid Tumors
Source: Front Immunol. 2022 Jul 6;13:905768. doi: 10.3389/fimmu.2022.905768 (PMC9296860; doi:10.3389/fimmu.2022.905768)
Supplement: Supplementary file 2 [file DataSheet_2.pdf]

Specimen ID	Age/Gender	Diagnosis	CA9 expression in GBM cells (%)	Survival time from diagnosis (months)
BT 935	53/F	P-GBM	66.80	8.3
BT 458	81/M	P-GBM	0.56	N/A
BT 428	63/F	P-GBM	69.76	13.8
BT 667	47/M	P-GBM	0.37	29.6
BT 566	55/F	R-GBM	0.33	N/A
BT 241	68/F	R-GBM	66.17	23.4
BT 618	67/F	R-GBM	13.58	N/A
BT 972	53/M	R-GBM	44.12	N/A
BT 799	77/F	P-GBM	0.15	6.2

Supplementary Table 1. GBM patient demographics. Related to Figures 1, 2, 5, 6, S2

Specimen ID	Gender	Age at diagnosis	Age at surgery	Diagnosis	CA9 expression (%)
RCC 22	F	66.8	67.6	RCC	_
RCC 162	_	_	_	RCC	61.80
RCC 243	_	_	_	RCC	80.50
RCC 323	M	57.9	57.10	RCC	48.70
RCC 364	M	45.7	45.9	RCC	_
RCC 407	M	62.7	62.9	RCC	_

Supplementary Table 2. RCC patient demographics. Related to Figures 3, 4, S2
